# Silencing microRNA-330-5p increases MMP1 expression and promotes an invasive phenotype in oesophageal adenocarcinoma

**DOI:** 10.1186/s12885-019-5996-3

**Published:** 2019-08-07

**Authors:** Becky A. S. Bibby, Cecelia S. Miranda, John V. Reynolds, Christopher J. Cawthorne, Stephen G. Maher

**Affiliations:** 1 Cancer Biology and Therapeutics Lab, School of Life Sciences, University of Hull, Hull, HU6 7RX UK; 2 Translational Radiobiology Group, Division of Cancer Sciences, University of Manchester, Manchester, M20 4GJ UK; 3 PET Imaging Centre, School of Life Sciences, University of Hull, Hull, HU6 7RX UK; 4 Biomedical Sciences, KU Leuven, Leuven, Belgium; 5 Trinity Translational Medicine Institute, Department of Surgery, Trinity College Dublin, St. James’s Hospital, Dublin 8, Ireland

**Keywords:** Oesophageal adenocarcinoma, microRNA, miR-330-5p, MMP1, Invasion, Chemoradiation therapy

## Abstract

**Background:**

Many patients diagnosed with oesophageal adenocarcinoma (OAC) present with advanced disease and approximately half present with metastatic disease. Patients with localised disease, who are managed with curative intent, frequently undergo neoadjuvant chemoradiotherapy. Unfortunately, ~ 70% of patients have little or no response to chemoradiotherapy. We previously identified miR-330-5p as being the most significantly downregulated microRNA in the pre-treatment OAC tumours of non-responders to treatment, but that loss of miR-330-5p had a limited impact on sensitivity to chemotherapy and radiation in vitro. Here, we further examined the impact of miR-330-5p loss on OAC biology.

**Methods:**

miR-330-5p was suppressed in OE33 OAC cells following stable transfection of a vector-driven anti-sense RNA. Whole transcriptome digital RNA-Seq was employed to identify miR-330-5p regulated genes, and qPCR was used for validation. Protein expression was assessed by protein array, Western blotting and zymography. Invasive potential was measured using a transwell assay system. Tumour xenograft growth profile studies were performed in immunocompromised CD1 mice.

**Results:**

In OE33 cells, suppression of miR-330-5p significantly altered expression of 42 genes, and several secreted proteases. MMP1 gene expression and protein secretion was significantly enhanced with miR-330-5p suppression. This corresponded to enhanced collagen invasion in vitro. In vivo, OE33-derived tumour xenografts with miR-330-5p suppression grew faster than controls.

**Conclusions:**

Loss of miR-330-5p expression in OAC tumours may influence tumour cell invasive capacity, tumour growth and therapeutic sensitivity via alterations to the tumour microenvironment.

**Electronic supplementary material:**

The online version of this article (10.1186/s12885-019-5996-3) contains supplementary material, which is available to authorized users.

## Background

More than half of patients diagnosed with oesophageal cancer will not survive more than a year and UK incidence rates are one of the highest in Europe [1]. Tumours are predominated by two histological subtypes, squamous cell carcinoma (SCC) and adenocarcinoma (OAC). In the past three decades a dramatic epidemiological shift in the incidence of these subtypes has occurred in both Europe and North America, with OAC now the predominant subtype, having increased more than 600% [2]. Even with advances in screening, diagnosis and treatment the overall 5-year survival rates have only risen from ~ 4% in the 1970s to ~ 15% at present, and currently reside at ~ 40% for localized disease [3].

OAC develops from the premalignant chronic acid reflux disease Barrett’s oesophagus (BO) [4]. The persistent exposure to low pH and bile acids causes a metaplastic transition from normal stratified squamous epithelium to single-layered mucin-secreting columnar epithelium. OAC is a disease of stepwise progression from non-dysplastic BO, to dysplastic BO and adenocarcinoma. However, the progression from BO to OAC occurs in less than 1% of patients and the majority of patients present with OAC without prior diagnosis of BO [5]. The biological drivers of OAC include chronic inflammation, disrupted cell adhesion, hypoxia and genomic instability [6–9].

Across most of Europe and North America a multimodal approach to treatment, involving neoadjuvant chemoradiotherapy (neo-CRT) prior to surgery, is generally recognized as the gold standard for managing locally advanced OAC [10]. Under the neo-CRT regimen the attainment of a complete or near complete pathological response, as dictated by the Mandard tumour regression grade (TRG), is a proxy for improved outcome for patients [11, 12]. Considering only ~ 30% of patients respond to neo-CRT treatment, the remaining ~ 70% are subjected to toxicity and are at increased risk of surgical complications with no apparent benefit and the prognosis of non-responders may be worsened due to the unnecessary delay to surgery [13]. Identifying those patients resistant to treatment through understanding the molecular and cellular basis governing response and resistance to neo-CRT is essential in improving treatment efficacy, increasing complete pathological response rates and ultimately OAC patient outcomes. Historically, the analysis of standard clinicopathological parameters is unable to predict tumour response to neo-CRT [14]. As patients with similar demographics, bearing tumours of similar clinical characteristics, can have vastly different responses to CRT it is likely that this dichotomy is due to subtle differences in the cellular and molecular environments of the tumours.

The current ‘omics’ era is providing large datasets from patient derived samples in an effort to identify disease drivers, tumour subtypes and biomarkers of disease progression and therapeutic response [15]. Mechanistic studies are needed alongside these ‘omics’ datasets to interpret the associated tumour biology. These studies will improve our understanding of tumour biology and support the development of new therapeutic approaches. Profiling of microRNAs (miRNAs), or miRnomics, has identified potential biomarkers and new therapeutic targets. MicroRNAs (miRNAs) are essential regulators of gene expression at the post-transcriptional level. They bind to complementary mRNA via non-stringent Watson-Crick base pairing and either repress protein translation or promote mRNA degradation [16]. Considering a single miRNA can target and regulate potentially thousands of mRNA this can dramatically alter the cellular protein expression landscape and signalling pathways, profoundly influencing cell behaviour. Genes encoding miRNAs have been mapped across the genome and are frequently encoded at fragile sites, hence they are susceptible to deletion and mutation [17]. Cancer associated miRNA are known as oncomiRs and can act as tumour suppressors or oncogenes [18]. MiRNAs have been demonstrated in many different cancers as functional modulators of chemosensitivity and radiosensitivity and are therefore promising biomarkers for the identification of patients with resistant tumours, as well as therapeutic targets for chemoradiation sensitisation [19].

There are a number of miRNAs that regulate sensitivity to chemotherapy and radiotherapy in OAC [20–23]. We have previously reported miR-330-5p as the most downregulated miRNA in OAC tumours of patients unresponsive to neo-CRT; however, miR-330-5p manipulation in vitro only had a modest impact on direct cellular radiosensitivity and no significant impact on chemosensitivity [20]. In more recent studies others have also identified miR-330 downregulation in multiple cancer types. In an oesophageal squamous cell carcinoma study, miR-330-3p was downregulated in neo-CRT non-responders [24]. In lung cancer patients with brain metastases downregulated miR-330 expression correlated with radiation sensitivity and poor prognosis [25].

In this current study the implications of miR-330-5p downregulation in OAC neo-CRT non-responders were further investigated. Firstly, transcriptome analysis was undertaken to identify gene expression changes associated with miR-330-5p silencing. The most significantly altered annotated target was MMP-1, and it was subsequently demonstrated in OE33 cells that MMP-1 expression is modulated by miR-330-5p and miR-330-5p suppression enhances cellular invasion. Furthermore, in vivo xenograft data demonstrated that silencing miR-330-5p expression enhances OAC tumour growth.

## Methods

### Cell lines and culture

The OE33 cell line was purchased from the ECACC (catalogue number 96070808). Cells were cultured in RPMI 1640 medium (Lonza, Switzerland) supplemented with 10% foetal bovine serum (Bio-Whittaker, Lonza, Switzerland), 1% penicillin/streptomycin (Lonza, Switzerland) and 1% GlutaMAX (Invitrogen, ThermoFisher Scientific, UK) as previously described [20]. Cell lines were regularly tested for mycoplasma contamination using the MycoAlert Mycoplasma Detection Kit (Lonza, Switzerland).

### Plasmid transfection

A miRNA-suppressing miR-ZIP plasmid was used for in vitro miR-330-5p suppression (catalogue number MZIP-330-5p-PA-1, System Biosciences, California, USA) as previously described [20]. Cells were transfected with the miR-ZIP plasmid or a scrambled non-targeting vector control MIRZIP-VC plasmid (catalogue number MZIP000-PA-1, System Biosciences) using Lipofectamine 2000 (Invitrogen, ThermoFisher Scientific, UK). The single clone (SC) cell line was derived from an individual clone that was selected after assessing GFP expression using fluorescent microscopy. The SC cell line had high levels of GFP expression indicating high expression of the miRZIP-330-5p plasmid. The miRZIP-VC SC was derived from an individual clone that was selected after assessing GFP expression using fluorescent microscopy. The heterogeneous clonal (HC) cell line was derived from a mixed population of stable clones. The miRZIP-VC HC was derived from a mixed population of stable clones. The miR-330 precursor plasmid was used for in vitro miR-330-3p/5p overexpression (catalogue number PMIRH330PA-1, System Biosciences). The miR-VC (catalogue number CD511B-1, System Biosciences) vector control plasmid was used as a control. Transient overexpression of miR-330-3p and miR-330-5p was confirmed via qPCR analysis, as previously described [20].

### RNA-seq whole transcriptome analysis

Total RNA was extracted from the OE33 miRZIP-330-5p SC and the OE33 miRZIP-VC SC. RNA-seq whole transcriptome analysis was outsourced to LC Sciences (Texas, USA). The RNA samples were prepared for shipping as advised by LC Sciences. LC Sciences performed whole transcriptome digital RNA-seq (DGE) using Illumina sequencing by synthesis technology, as previously described [23].

### RNA extraction and qPCR

Total RNA extraction, RNA quantification, reverse transcription and qPCR were performed as previously described [20]. For qPCR, QuantiTect Primer Assays were used for MMP1, MMP7 and B2M (Catalogue numbers; QT00014581, QT00001456 and QT00088935, respectively) (Qiagen). Relative MMP1 or MMP7 mRNA expressions were determined using the 2^-ΔΔCt^ (Livak) method [26].

### Preparation of conditioned media

In 6 cm dishes, 8 × 10^5^ cells were seeded in complete medium and incubated for 48 h to reach ~ 70% confluency. The medium was discarded and cells were washed with PBS before 2.5 mL serum free RPMI 1640 was applied. Cells were incubated for 24 h, and then the conditioned medium was subsequently harvested and centrifuged at 4 °C for 5 min at 300×*g* to pellet non-adherent cells and debris. The conditioned medium was then transferred into a centrifugal filter column (5 kDa molecular weight cut-off) to concentrate the protein (Vivaspin® 4 Sartorius, ThermoFisher, UK). Columns were centrifuged at 4 °C for 60–70 min at 4000×*g* to concentrate conditioned medium. Approximately 100 μL of concentrated protein sample was recovered.

### Western blotting

The BCA assay (Pierce, Thermo Scientific, UK) was used to quantify protein content in the concentrated conditioned media, and 50 μg of protein was loaded onto 10 or 12% SDS-PAGE gels. Electrophoretically separated proteins were transferred onto PVDF (ThermoFisher Scientific, UK) using a wet transfer tank system (BioRad, UK). Following transfer, PVDF membranes were blocked with 5% non-fat milk TBST (0.1% Tween) solution. Blots were probed for MMP1 (1:1000 dilution, MAB901 mouse monoclonal, R and D Systems, UK), MMP7 (1:1000 dilution, MAB9071 mouse monoclonal, R and D Systems, UK) and the loading control β-actin (1:10000 dilution, AC-15 mouse monoclonal, Santa Cruz Biotechnology, Texas, USA). Image Lab 3.0 software (BioRad, UK) was used for densitometry analysis of western blots.

### Zymography

Gelatin zymography was employed to detect the presence and activity of MMP1 in conditioned serum free media. Samples were prepared by combining 20 μL of concentrated conditioned medium with 5 μL of non-reducing sample buffer. The prepared samples were loaded into the wells of a 1 mg/mL gelatin gel and proteins were separated using electrophoreses (120 V for 2 h). Gels were transferred into renaturing wash buffer (2.5% Triton-X, 50 mM Tris pH 7.4, 5 mM CaCl_2_) for 1 h, during which time the buffer was changed three times. The zymogram was rinsed in deionised water and incubated in developing buffer (50 mM Tris, pH 7.4, 5 mM CaCl_2_) at 37 °C overnight. Zymograms were stained with coomassie stain (0.125% w/v coomassie brilliant blue R-250, 1% v/v acetic acid, 45% v/v ethanol, 54% v/v water) for 1 h and destained with solution I (62.5% v/v ethanol, 25% v/v acetic acid, 12.5% v/v water) for 30 min and solution II (0.05% v/v ethanol, 7% v/v acetic acid, 92.95% v/v water) for 1 h. Zymograms were washed with water for 30 min and stored in gel preservative solution (3% v/v glycerol, 30% v/v methanol, 67% v/v water). Zymograms were imaged using the Molecular Imager ChemiDoc XRS with Image Lab 3.0 software (BioRad, UK). The images appear as a ‘reverse’ coomassie, with the zymogram staining gelatin blue and the hydrolase activity of the enzyme visible as a white band/cleared area.

### Protease and protease inhibitor antibody arrays

Antibody-based arrays were used to assess the relative expression levels of 32 proteases and 35 protease inhibitors in conditioned medium (75 μg protein) as per the manufacturer’s instructions (Proteome Profiler antibody arrays, R and D Systems, UK).

### Transwell invasion assay

The Corning BioCoat Growth Factor Reduced matrigel Invasion Chamber (8 μm membrane) assay was used to measure cellular invasion, as per the manufactures instructions (VWR, UK). Cells were seeded at a density of 2.5 × 10^4^ cells per insert and plates were incubated for 24 h. Membranes were mounted onto glass sides with mounting media containing DAPI stain (ProLong Gold Antifade Mountant with DAPI, Invitrogen, UK). Slides were visualised under the microscope using the DAPI filter and × 10 magnification. The DAPI stained nuclei were counted using Image J software.

The colorimetric OCM high sensitivity non-cross-linked collagen invasion assay was used to determine cellular invasiveness, as per the manufacturer’s instructions (Merck Millipore, Darmstadt, Germany). Briefly, 2.5 × 10^5^ cells in serum-free medium were applied to the collagen coated upper chamber inserts and plates were incubated at 37 °C in a humidified CO_2_ incubator for 24 or 48 h. Following processing and staining as per the manufacturer’s instructions, stained inserts were incubated in extraction buffer (provided) for 15 min, and subsequently optical density at 560 nm was measured.

### In vivo xenografts

OE33 miRZIP-330-5p HC and miRZIP-VC HC cells were prepared for subcutaneous injection into CD1 nude mice. For each cell line, 6 mice were implanted subcutaneously on the right flank with 4 × 10^6^ cells/100 μl in 50% serum-free media/50% Cultrex (RnD Systems, UK) as described [27]. Tumour measurements were taken 2–4 times per week using callipers. When tumours were at size, animals were sacrificed via cervical dislocation. All animal procedures were approved by the University of Hull Animal Welfare Ethical Review Body and carried out in accordance with the United Kingdom’s Guidance on the Operation of Animals (Scientific Procedures) Act 1986 and within guidelines set out by the United Kingdom National Cancer Research Institute Committee on the Welfare of Animals in Cancer Research [28] under Home Office Project License number 60/4549 held by Dr. Cawthorne.

### Statistical analysis

Unless otherwise stated data are presented as the mean ± standard error of the mean (SEM) and are representative of at least three independent experiments. Statistical analysis was carried out using GraphPad InStat v3. Specific statistical tests used are disclosed in the relevant figure legends. Differences were considered to be statistically significant at *p* < 0.05.

## Results

### Identifying the gene targets of miR-330-5p

Endogenous miR-330-5p expression in OE33 OAC cells was silenced using the miRZIP-330-5p vector, which produces an anti-sense miR-330-5p that irreversibly binds to endogenous miR-330-5p, thereby inhibiting its function. Two stable OE33 miRZIP-330-5p models were established; a single clone model (SC) and a heterogeneous clonal (HC) model. To identify targets and pathways that were altered by miR-330-5p silencing the miRZIP-330-5p SC model was used for transcriptome/DGE analysis. Forty-two genes were differentially expressed between the OE33 miRZIP-VC SC and the OE33 miRZIP-330-5p SC cell lines (Additional file 1). Of these, 19% were upregulated (8 genes) and 81% were downregulated (34 genes) as a result of miR-330-5p silencing.

### Validating MMP1 as a target of miR-330-5p

**Fig. 1:**
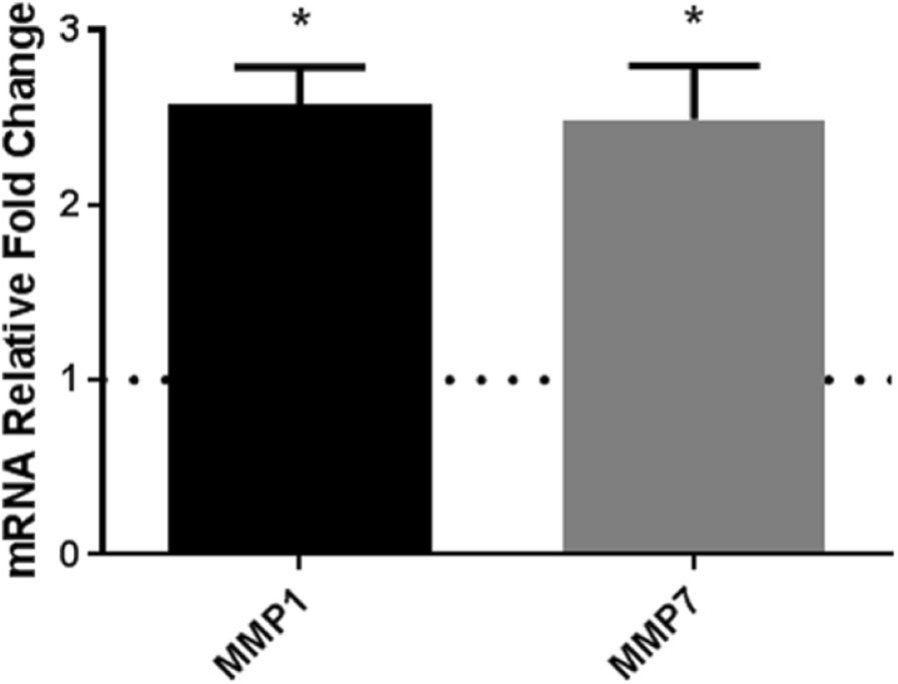
Silencing miR-330-5p in OE33 cells increases *MMP1* and *MMP7* mRNA expression. In the OE33 miRZIP-330-5p SC qPCR analysis confirmed a ~ 2.5 fold increase in *MMP1* and *MMP7* mRNA expressions. The relative fold change in *MMP1* and *MMP7* in the miRZIP-330-5p cell line was calculated relative to the miRZIP-VC SC control (normalised to 1, dotted line). Data are presented as the mean ± SEM (*n* = 3). Statistical analysis was performed using the one-sample *t*-test; * *p* < 0.05

**Fig. 2:**
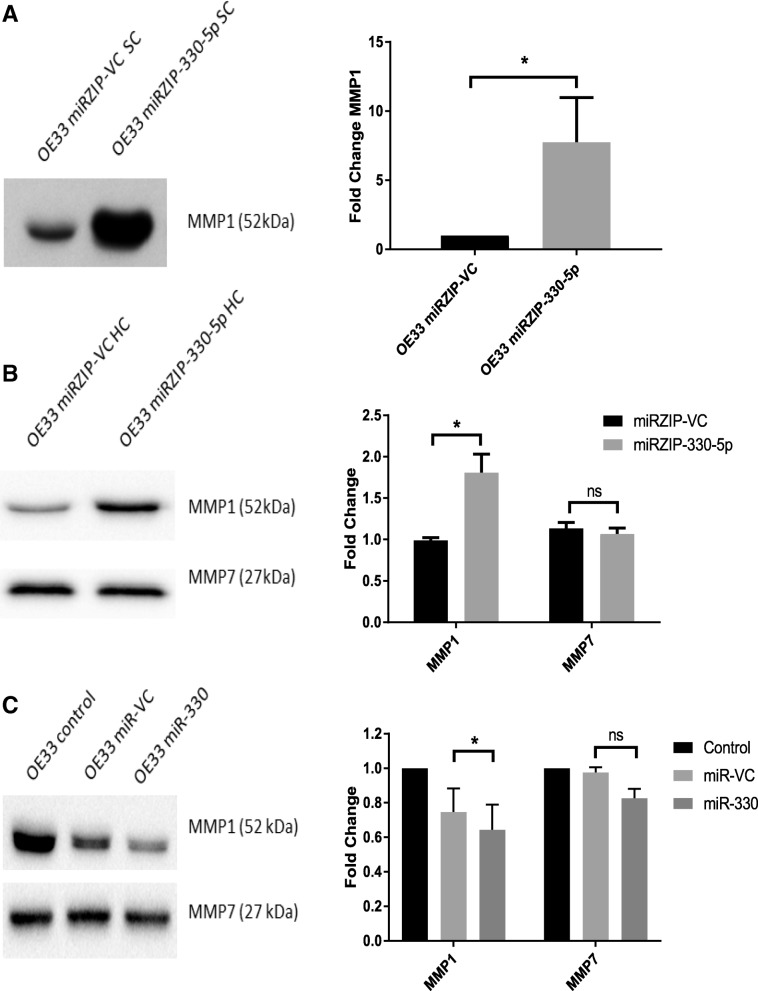
MiR-330-5p regulates the expression of extracellular MMP1 protein expression. **a** MMP1 protein expression in conditioned media was increased in the OE33 miRZIP-330-5p SC compared to the miRZIP-VC SC. The blot is representative of *n* = 3 independent experiments. Statistical analysis was performed using densitometry data and a one-tailed unpaired *t*-test; * *p* < 0.05. **b** MMP1 protein expression in conditioned media was increased in the OE33 miRZIP-330-5p HC compared to the miRZIP-VC HC. The expression of MMP7 protein was not increased by miR-330-5p silencing. The blot is representative of *n* = 3 independent experiments. Statistical analysis was performed using densitometry data and the unpaired *t*-test; * *p* < 0.05; ns, not significant. **c** In the OE33 cell line the transient overexpression of miR-330 significantly decreased the expression of MMP1 protein in the 24 h conditioned media (48 h post-transfection) compared to the miR-VC. Blots represent *n* = 3 independent experiments. Statistical analysis was performed using densitometry data and the paired *t*-test; * *p* < 0.05; ns, not significant

The DGE analysis identified a 5-fold increase in *MMP1* and a 2.5-fold increase in *MMP7* in the miRZIP-330-5p SC, which was validated via qPCR (Fig. 1). The increase in the *MMP1* mRNA corresponded with an increase in MMP1 protein expression in conditioned media from both the miRZIP-330-5p SC and miRZIP-330-5p HC cell lines (Fig. 2a and b). There was no change in MMP7 protein expression despite the increase in mRNA expression (Fig. 2b), and this was subsequently used as a protein loading control. The upregulated expression of MMP1 corresponded to an increase in the expression of pro-MMP1 and active MMP1 protein (Additional file 2).

Of the eight upregulated genes identified in the DGE, four genes have potential binding sites for miR-330-5p (Additional file 1) [29]. The *MMP1* mRNA has three predicted binding sites for miR-330-5p and it was hypothesised that miR-330-5p likely directly targets the MMP1 mRNA. Concordantly, the transient overexpression of miR-330 in the OE33 cell line decreased extracellular MMP1 protein expression (Fig. 2c).

**Fig. 3:**
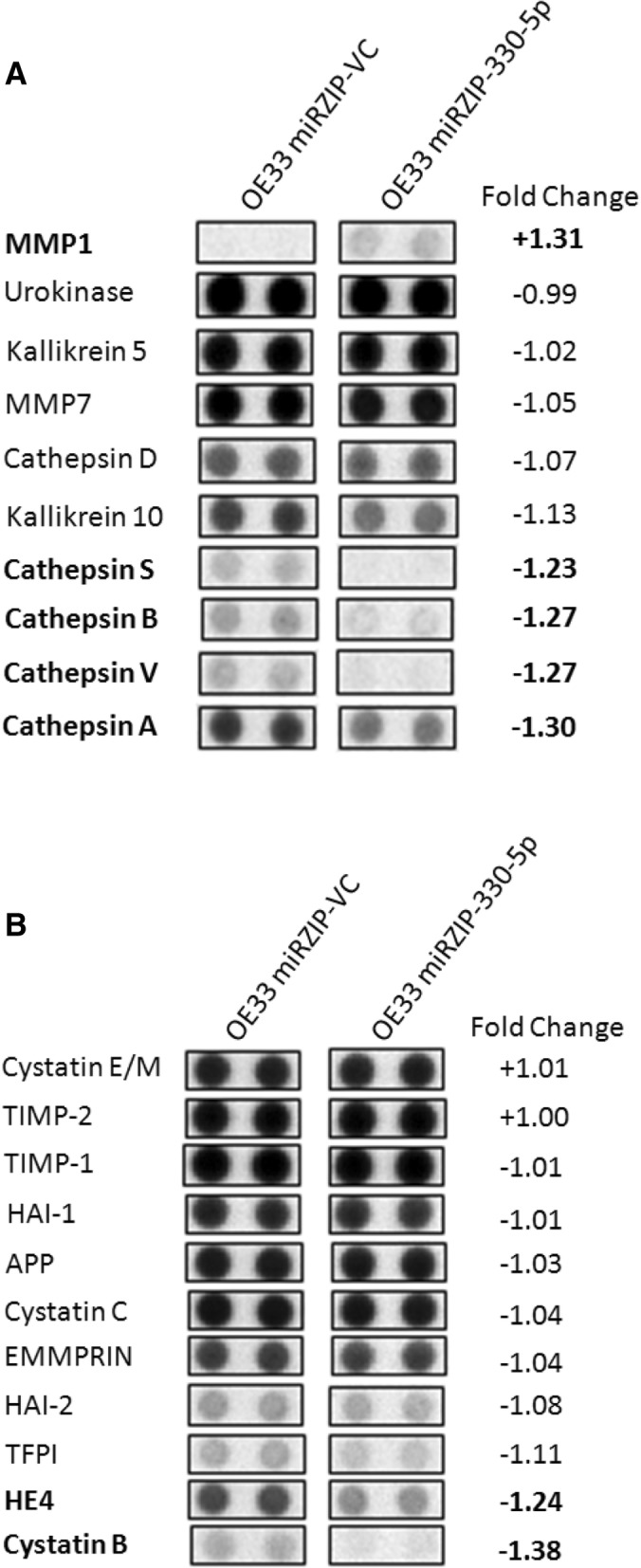
Protease and protease inhibitor antibody-based array profiles. Silencing miR-330-5p altered the expression of secreted proteases (**a**) and protease inhibitors (**b**) in 24 h conditioned media. Densitometry analysis was used to calculate the fold change in protein expression in the OE33 miRZIP-330-5p SC relative to the OE33 miRZIP-VC SC. Highlighted in bold are proteins that exceeded ±1.2 fold change. The antibody-based array confirmed an increase in MMP1 expression with miR-330-5p silencing, and confirmed no increase in MMP7 expression. Data represent a single experimental repeat

Antibody-based arrays were used to analyse the expressions of 32 proteases and 35 protease inhibitors in conditioned media from the miRZIP-VC SC and the miRZIP-330-5p SC cell lines (Fig. 3a and b). The antibody arrays further supported the previous observations of increased MMP1 and unaltered MMP7 protein expression in the conditioned media of the miRZIP-330-5p cells. This suggested miR-330-5p regulates MMP1 protein expression and the biological implications of this relationship were further investigated.

### Silencing miR-330-5p increased MMP1 expression and altered invasive potential

**Fig. 4:**
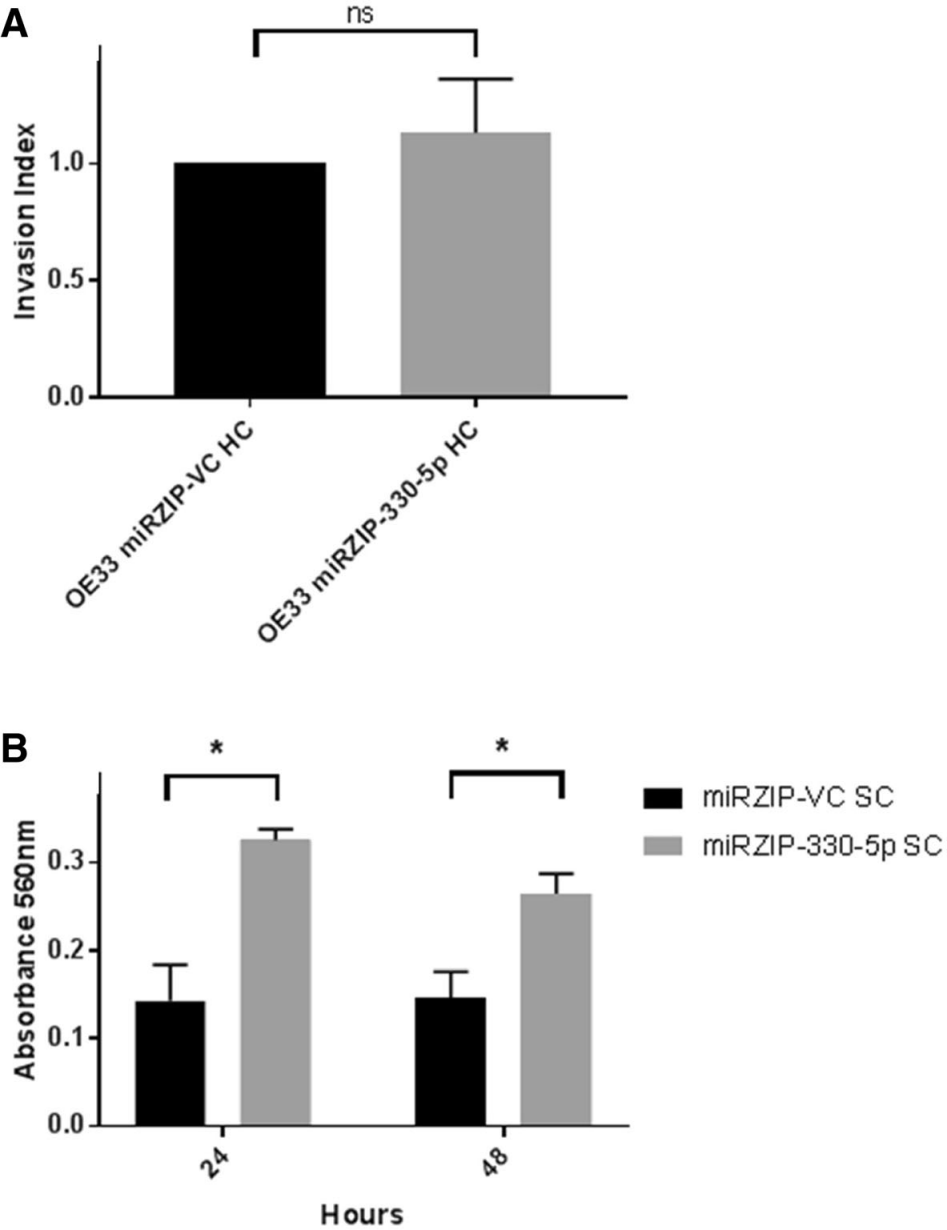
Silencing miR-330-5p enhances OE33 cell invasion. **a** The invasive potential of the OE33 miRZIP-330-5p HC was not significantly increased relative to the miRZIP-VC HC in the 24 h matrigel invasion assay. Data are representative of *n* = 3 independent experiments. Data presented as the mean ± SEM. Statistical analysis was performed using the one-sample *t*-test; ns, not significant. However, in (**b**) the invasive potential of the OE33 miRZIP-330-5p SC was significantly increased relative to the miRZIP-VC SC in the more sensitive non-cross-linked collagen invasion assay at 24 h and 48 h. Data are representative of *n* = 3 independent experiments. Data presented as the mean ± SEM. Statistical analysis was performed using the paired *t*-test; * *p* < 0.05

The matrix metalloproteinase family are most commonly associated with remodelling of the extracellular matrix and cellular invasion. Therefore, the invasive potential of the miRZIP-330-5p HC cell line compared to the miRZIP-VC HC cell line was examined. Despite the increase in MMP1 protein expression with miR-330-5p silencing, the OE33 miRZIP-330-5p HC cell line did not display a more invasive phenotype at the time points tested in the matrigel-based transwell invasion assay (Fig. 4a). However, OE33 are considered poorly invasive in cross-linked collagen, and inclusion of the OE33 miRZIP-330-5p SC cell line in a non-cross linked collagen transwell invasion assay demonstrated significantly enhanced invasive potential at 24 h and 48 h (Fig. 4b).

### miR-330-5p silencing accelerates in vivo tumour growth

**Fig. 5:**
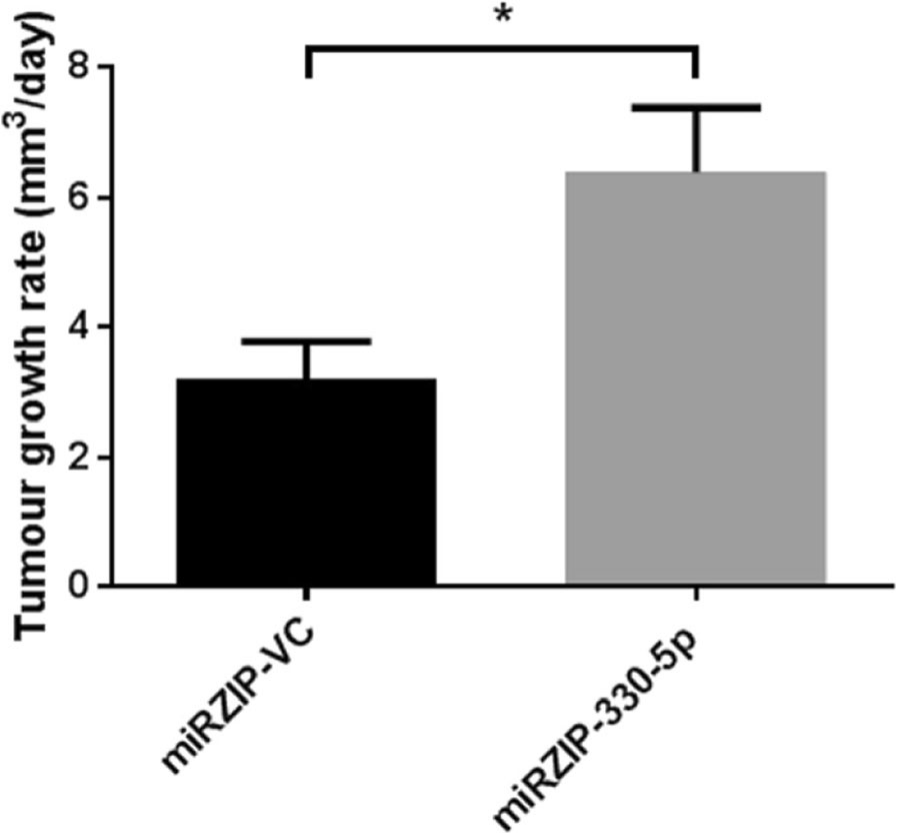
Growth profiles of tumour xenografts established from OE33 miRZIP-VC and miRZIP-330-5p heterogeneous cell lines. (A) Mice were implanted on day 0 and tumour growth rate in mm^3^ per day was calculated between days 18 and 46. These measurements were taken before tumour sizes exceeded 200 mm^3^. Animals per group: miRZIP-VC *n* = 5, miRZIP-330-5p *n* = 4. Data are presented as the mean ± SEM. Statistical analysis was performed using the unpaired *t*-test; *p < 0.05

The OE33 miRZIP-VC HC and miRZIP-330-5p HC cell lines were used to establish in vivo tumour xenografts in CD1 mice. Tumour growth profiles indicated significantly accelerated tumour growth in the miRZIP-330-5p xenografts compared to the miRZIP-VC xenografts (Fig. 5).

## Discussion

We previously demonstrated in pre-treatment tumour biopsies from OAC neo-CRT non-responders that miR-330-5p was the most significantly downregulated miRNA [20]. In vitro miR-330 overexpression and miR-330-5p silencing did not alter cellular sensitivity to cisplatin or 5-FU but miR-330-5p silencing marginally increased radioresistance [20]. To further study the biological significance of downregulated miR-330-5p in OAC the expression of miR-330-5p was silenced in the OE33 cell line using a plasmid vector encoding the anti-sense miR-330-5p, to effectively mimic the downregulated miR-330-5p expression observed in the tumours of neo-CRT non-responders.

Gene expression analysis was used to identify potential direct and indirect targets of miR-330-5p; mRNA targets of miR-330-5p that are translationally repressed by mechanism other than degradation are unlikely to have altered mRNA expression as a result of miR-330-5p silencing. The majority of gene expression changes reported here are most likely to be indirectly associated with miR-330-5p silencing. There were 8 genes (7 annotated) that were upregulated in response to miR-330-5p silencing. The most upregulated gene was *PRAME* (*preferentially expressed antigen of melanoma*). PRAME is a tumour antigen that induces a cytotoxic T-cell immune response [30]. Although the tumour antigen is preferentially expressed in melanoma it has also been identified in a number of other cancers and correlates with prognosis and survival [31]. The second most upregulated gene with miR-330-5p silencing was *ADRA2C* (*adrenoreceptor alpha 2C*), which has a predicted binding site for miR-330-5p [29]. Expression of alpha-2-adrenergic receptors has been reported in breast cancer cells and tissue, and receptor activation induces proliferation [32]. It is possible that ADRA2C may be functionally involved in the accelerated tumour growth observed in vivo in this present study. In colorectal cancer *ADRA2C* gene expression has been identified as a predictor of advanced clinical stage [33]. The third and fourth most upregulated genes were *MMP1* and *MMP7*.

The upregulated expressions of *MMP1* and *MMP7* with miR-330-5p silencing were of particular interest because MMP1 and MMP7 have previously been reported as prognostic markers in oesophageal cancer [34–36]. The first of these studies reported MMP1 as an independent prognostic marker in a cohort of 19 SCC and 27 OAC patients [34]. Survival analysis showed the MMP1 positive group had a median survival of 7 months compared to 16 months in the MMP1 negative group [34], suggesting MMP1 overexpression promotes a poor prognosis. The role of MMP1 in early disease was reported in another study that identified MMP1 as a pre-invasive factor in Barrett’s oesophagus-associated OAC [37]. The expression of MMP1 was confirmed in 95% of patients with OAC and Barrett’s oesophagus, furthermore, in vitro MMP1 expression strongly correlated with proliferation. Although MMP1 expression was not associated with overall survival, high expression of MMP1 was associated with lymph node metastasis [37]. The upregulation of MMP1 expression in OAC has been linked to the EST-domain transcription factor PEA3 subfamily, which promotes MMP1 expression and potentially drives metastasis [38]. The MMP family degrade various components of the extracellular matrix and enable cancer cells to invade and metastasise. However, the activities of MMPs are not limited to extracellular matrix remodelling [39]. Recently, MMP1 has been implicated as a promoter of angiogenesis and in the context of tumour sensitivity to CRT, neovascularisation, vascularity and hypoxia are all factors that significantly influence tumour response to therapy [40].

The upregulated expression of the *MMP1* mRNA corresponded to an increase in the expression of pro-MMP1 and active MMP1 protein (Additional file 2). Conversely, the increase in the *MMP7* mRNA did not correspond to an increase in the MMP7 protein. The increase in MMP1 protein expression was far greater in the miRZIP-330-5p SC than the in the miRZIP-330-5p HC. It is likely that the silencing of miR-330-5p in HC cell line was not as extensive as it was in the SC cell line and this may explain the difference in MMP1 expression between the cell lines. There are three possible binding sites for miR-330-5p in the *MMP1* mRNA, and no predicted binding sites for miR-330-3p, suggesting miR-330-5p may specifically target and regulate *MMP1* expression [29]. In addition, the overexpression of miR-330 decreased the expression of MMP1 further supporting the role of miR-330-5p as a repressor of *MMP1* translation.

In the non-crossed linked collagen assay the invasive potential of the miRZIP-330-5p SC was significant enhanced compared to the miRZIP-VC SC. However, invasion was not enhanced in the 24 h matrigel assay. The preferred substrate of MMP1 is collagen and this may in part explain the different results from the two invasion assays. Furthermore the increase in MMP1 expression was more subtle in the miRZIP-330-5p HC used in the matrigel assay compared to the miRZIP-330-5p SC used in the collagen assay. This study is not the first to identify miR-330-5p as a modulator of cellular invasion. The in vitro overexpression of miR-330-5p in cutaneous malignant melanoma has been shown to decrease cellular migration and invasion [41]. In non-small cell lung cancer miR-330-5p was found to be downregulated and restoring expression in vitro inhibited cell growth and promoted apoptosis [42]. Another non-small cell lung cancer study identified the long non-coding RNA (lncRNA) PCAT6 (prostate cancer-associated transcript 6) as a promoter of migration and invasion though regulation of miR-330-5p [43].

Considering that we had originally identified miR-330-5p downregulation in patient tumour biopsies, an in vivo model was established using the miRZIP-330-5p cells. The mixed population of clones (miRZIP-330-5p HC and miRZIP-VC HC) were considered to be a more relevant model of tumour heterogeneity than the cell lines derived from a single clone. In vivo OE33 miRZIP-330-5p HC xenografts grew significantly faster than the miRZIP-VC HC xenografts. The tumour xenografts include elements of an intact tumour microenvironment, such as stroma and vasculature that cannot be accounted for in vitro. However, the in vivo model also has limitations and was not suitable for studying potential changes in invasion, typically because the subcutaneous xenografts were established in immune-compromised mice using a relatively non-invasive cell line. In spite of these limitations, the enhanced tumour growth observed in vivo demonstrates that silencing a single miRNA can have a significant impact on OAC tumour biology.

## Conclusion

In summary, the data support the role of miR-330-5p as a modulator of MMP1 expression. Silencing miR-330-5p in vitro increased MMP1 expression and enhanced invasive potential. In OAC tumours downregulated miR-330-5p was associated with CRT resistance [20]. Considering miRNA are produced in all cell types and are known to directly and indirectly modulate the tumour microenvironment, therapeutic intervention at the miRNA level could alter the biology of the extracellular microenvironment and CRT sensitivity [44]. It is not known if downregulated miR-330-5p is associated with enhanced MMP1 expression in OAC tumours although silencing miR-330-5p in vivo enhanced tumour growth. Considering miR-330-5p did not significantly alter cellular response to CRT in our previous in vitro study, the identification of miR-330-5p regulated genes and proteins with extracellular functions was of particular interest here. Downregulated miR-330-5p expression in OAC tumours could confer a more invasive and aggressive tumour phenotype that indirectly confers resistance to CRT.

## Additional files

Additional file 1: **Table S1.** Transcriptome gene expression analysis log_2_ fold change in the OE33 miRZIP-330-5p SC cell line and predicted binding sites for miR-330-3p and miR-330-5p. (DOCX 17 kb)

Additional file 2: **Figure S1.** Silencing miR-330-5p increased the expression of the inactive and active MMP1 protein isoforms. Gelatin zymography confirmed an increase in MMP1 protein expression. The expression of the inactive pro-MMP1 and active MMP1 were both increased in the 24 h conditioned media from the OE33 miRZIP-330-5p SC compared to the miRZIP-VC SC. Blot is representative of *n* = 3 independent experiments. (DOCX 260 kb)

## Data Availability

The authors declare that all the data supporting the findings of this study are available within the article and additional files.

## Abbreviations

HC: Heterogeneous clone
miRNA/miR: microRNA
MMP: Matrix metalloproteinase
neo-CRT: Neoadjuvant chemoradiotherapy
neo-CT: Neoadjuvant chemotherapy
OAC: Oesophageal adenocarcinoma
SC: Single clone model
SCC: Squamous cell carcinoma
TRG: Tumour regression grade
