# Supplementary material for: Silencing microRNA-330-5p increases MMP1 expression and promotes an invasive phenotype in oesophageal adenocarcinoma
Source: BMC Cancer. 2019 Aug 7;19:784. doi: 10.1186/s12885-019-5996-3 (PMC6686260; doi:10.1186/s12885-019-5996-3)
Supplement: Supplementary file 1 — Table S1. Transcriptome gene expression analysis log2 fold change in the OE33 miRZIP-330-5p SC cell line and predicted binding sites for miR-330-3p and miR-330-5p. (DOCX 17 kb) [file 12885_2019_5996_MOESM1_ESM.docx]

**Table S1.** Transcriptome gene expression analysis log_2_ fold change in the OE33 miRZIP-330-5p SC cell line and predicted binding sites for miR-330-3p and miR-330-5p.

| **Gene** | **Fold Change** | **Predicted miR-330-5p binding site?** | **Predicted miR-330-3p binding site?** |
| --- | --- | --- | --- |
| * | INF |  |  |
| PRAME | INF |  |  |
| ADRA2C | INF | Y |  |
| MMP1 | 5.39 | Y |  |
| MMP7 | 2.63 |  |  |
| DPP4 | 2.60 |  | Y |
| AQP3 | 2.11 | Y |  |
| PVRL1 | 1.65 | Y | Y |
| NR4A1 | -1.52 | Y |  |
| SLFN5 | -1.55 |  |  |
| * | -1.63 |  |  |
| DDX60 | -1.73 |  | Y |
| COL17A1 | -1.80 | Y |  |
| COMMD10 | -1.82 |  | Y |
| IFIT3 | -1.83 |  | Y |
| S100P | -1.88 | Y |  |
| LCN2 | -1.90 |  |  |
| SAMD9 | -1.90 |  |  |
| ISG15 | -1.90 |  |  |
| SAMD9L | -1.96 | Y | Y |
| FST | -1.96 |  |  |
| IFI27 | -2.17 | Y |  |
| IRF7 | -2.30 |  |  |
| LAMP3 | -2.30 | Y | Y |
| LOC644100 | -2.52 |  |  |
| WFDC2 | -2.62 |  |  |
| HIST2H2BE | -2.69 |  |  |
| CMPK2 | -2.70 |  | Y |
| RCAN1 | -2.70 |  | Y |
| MX1 | -2.74 |  |  |
| IFIT1 | -2.86 |  |  |
| IFITM1 | -2.86 |  |  |
| IFI6 | -2.96 | Y |  |
| SLC6A14 | -3.04 |  |  |
| HERC5 | -3.11 |  |  |
| OAS2 | -3.42 |  |  |
| IFI44L | -3.78 | Y | Y |
| MX2 | -3.82 | Y | Y |
| * | -INF |  |  |
| * | -INF |  |  |
| * | -INF |  |  |
| STS | -INF | Y | Y |

**Novel non-annotated gene; INF, infinity; Y, yes*
