# Supplementary figures and images for: Silencing microRNA-330-5p increases MMP1 expression and promotes an invasive phenotype in oesophageal adenocarcinoma
Source: BMC Cancer. 2019 Aug 7;19:784. doi: 10.1186/s12885-019-5996-3 (PMC6686260; doi:10.1186/s12885-019-5996-3)

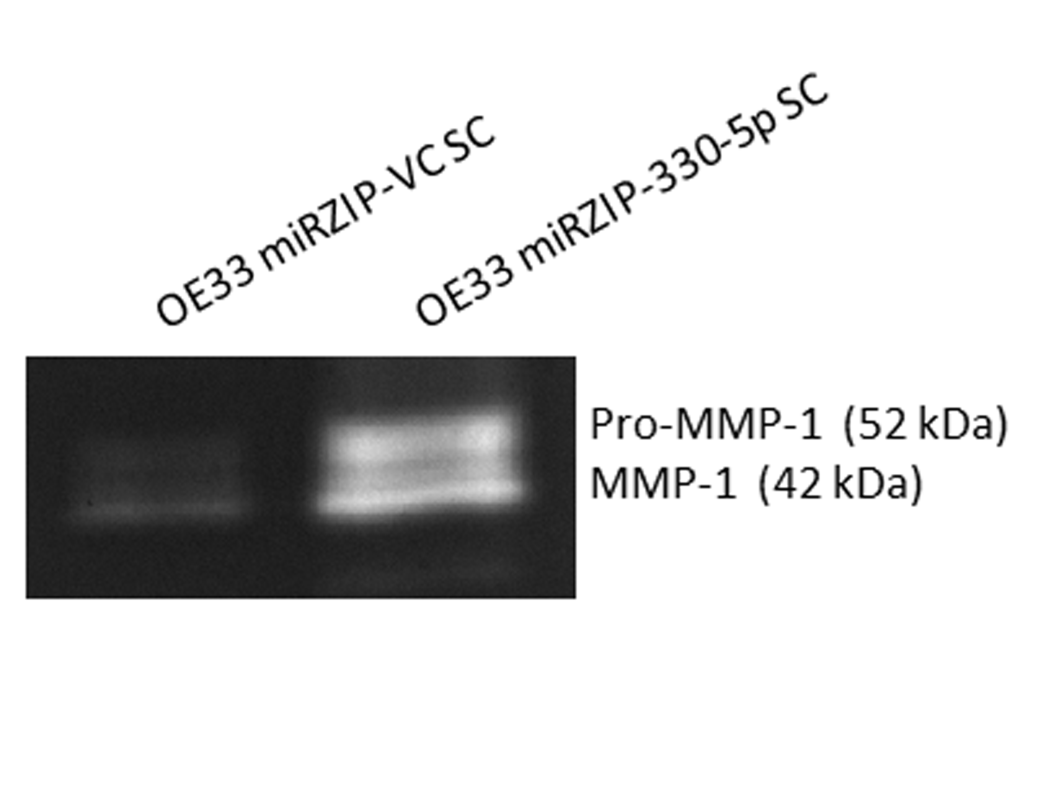

Supplement: Supplementary file 2 — Figure S1. Silencing miR-330-5p increased the expression of the inactive and active MMP1 protein isoforms. Gelatin zymography confirmed an increase in MMP1 protein expression. The expression of the inactive pro-MMP1 and active MMP1 were both increased in the 24 h conditioned media from the OE33 miRZIP-330-5p SC compared to the miRZIP-VC SC. Blot is representative of n = 3 independent experiments. (DOCX 260 kb) [file 12885_2019_5996_MOESM2_ESM.docx]
